# Use of intravenous and oral sodium thiosulfate for acute treatment and chronic management of nonuremic calciphylaxis: A retrospective case series

**DOI:** 10.1016/j.jdcr.2026.01.050

**Published:** 2026-02-06

**Authors:** Benjamin Zhu, Joseph C. English

**Affiliations:** a University of Pittsburgh, School of Medicine, Pittsburgh, Pennsylvania; b Department of Dermatology, University of Pittsburgh, Pittsburgh, Pennsylvania

**Keywords:** calciphylaxis, non-nephrogenic, non-uremic calciphylaxis, sodium thiosulfate, wound healing

## Introduction

Calciphylaxis is a rare, painful, and life-threatening syndrome characterized by calcification, thrombosis, and occlusion of small to medium sized cutaneous arteries leading to skin necrosis and ulceration.^1^ Calciphylaxis is further subcategorized into uremic and non-uremic types, which have similar clinical and histopathological features but differ in patient populations, prognosis, and risk factors. Uremic calciphylaxis occurs primarily in patients with end-stage renal disease (ESRD) on dialysis and has a worse prognosis (1-year mortality 45% to 80%).^1^ Its associated skin lesions are centrally located to abdominal skin and acral locations. These patients are typically primarily managed by nephrologists. In contrast, non-uremic calciphylaxis (NUC) is commonly seen in patients with obesity, primary hyperthyroidism, autoimmune disorders, vitamin D/K deficiency, liver disease, diabetes, malignancy, or warfarin or prednisone use. It is also rarer, associated with predominantly acral lesions, occurs in patients without end stage renal disease, and primarily managed by dermatologists.^2^ One year mortality for NUC is high (25% to 45%), but less than uremic calciphylaxis.^1^

The rarity of calciphylaxis often creates a therapeutic challenge, as established treatment protocols remain scarce. Aggressive wound care, including debridement of necrotic tissue and multimodal pain control, is essential. Sepsis from wounds is a leading cause of mortality.^1^^,^^3^ Predictors of increased calciphylaxis mortality include lesional distribution, atrial fibrillation, and being on dialysis at diagnosis.^4^ Pharmacologically, sodium thiosulfate (STS) has emerged as a therapeutic option, due to its proposed mechanisms involving calcium chelation, antioxidant properties, and vasodilation.^5^ Intravenous (IV) STS is used off label for treating calciphylaxis, with the most common regimen being 25 g administered intravenously over 30 to 60 minutes, typically 3 times per week for 36 treatments, though dosing and duration are not standardized and should be individualized based on clinical factors such as response and tolerability. Potential side effects of STS therapy include metabolic acidosis, volume overload, hypocalcemia, and QTc prolongation. These side effects are more common with IV administration and in patients with underlying cardiac or metabolic comorbidities.

However, IV STS is associated with high costs due to the need for infusion facilities. Per-infusion charges vary across health systems but the typical cost of a multi-month course can range in the thousands to tens of thousands of dollars.^6^^,^^7^ Oral STS may offer a lower-cost (compounded by ChemistryRx, 750 mg twice daily, $86 for 1-month supply), more accessible option for long-term maintenance; however, formal cost-effectiveness analyses are required to confirm its value.

Additionally, the role of STS remains underexplored in the treatment of NUC as most of the aforementioned examples focus on calciphylaxis associated with ESRD.^8^ As nephrologists are less involved in the management of non-uremic cases, it is critical that dermatologists understand how to manage NUC and use potential treatment options. In this case series, we report 4 cases of resolution of acute NUC flares treated with IV STS, with sustained suppression through oral STS maintenance therapy managed by their dermatologist.

**Fig 1:**
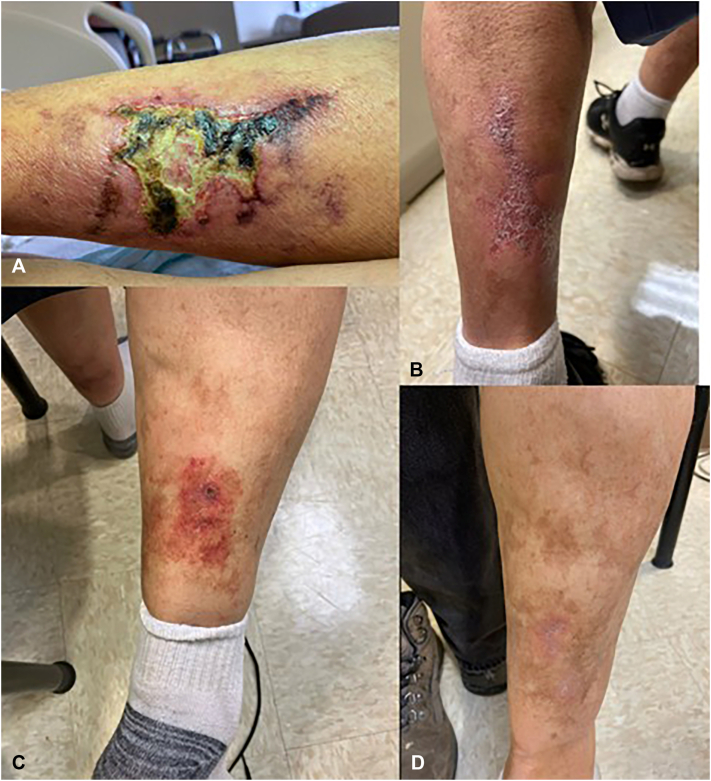
Skin lesions at prior to treatment initiation with IV STS (**A**), 36 IV treatments (**B**), skin lesions at flare (**C**), 72 IV treatments and oral maintenance (**D**), case 1.

**Fig 2:**
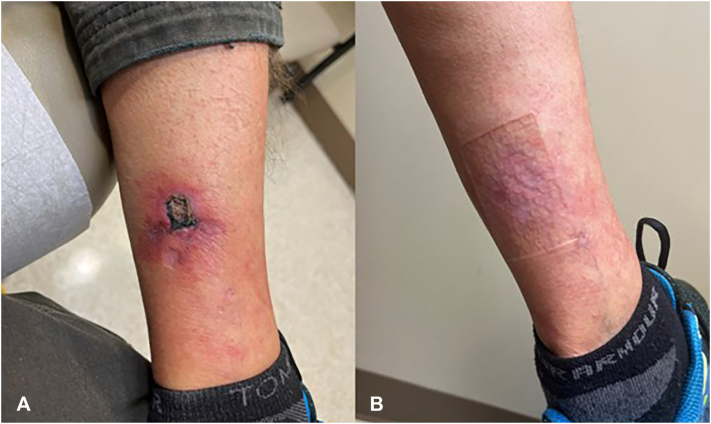
Skin lesions at biopsy (**A**), 36 treatments, IV STS, 25g (**B**), case 2.

**Fig 3:**
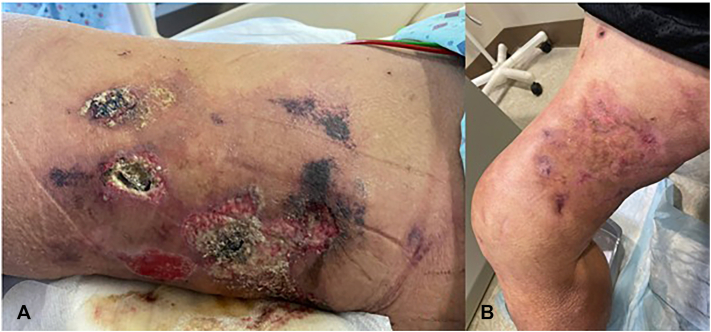
Skin lesions at prior to treatment initiation with IV STS (**A**), 108/108 IV treatments (**B**), case 3.

**Fig 4:**
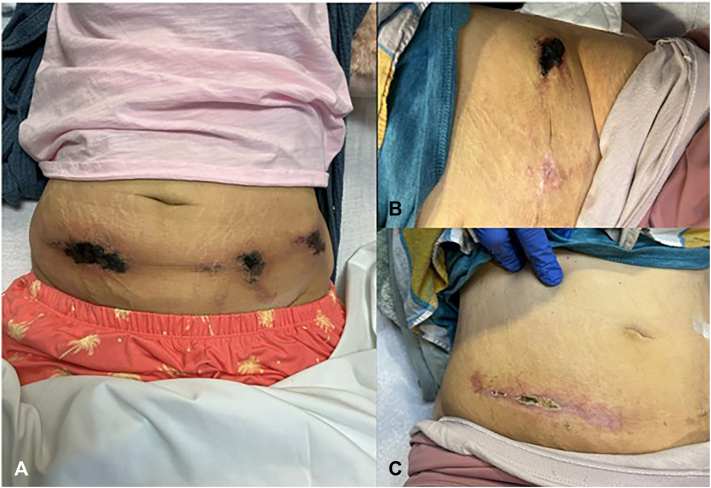
Skin lesions at prior to treatment initiation with IV STS (**A**), 17 IV treatments (**B**), case 4.

We reviewed 4 biopsy-confirmed NUC cases treated at a large academic medical center from December 2022 to January 2026. All patients received IV STS as initial therapy (25 g 2 or 3 times weekly, 36 infusions per course) with weekly labs and EKGs. Additional IV STS courses were administered for persistent or recurrent disease. Afterwards, patients were transitioned to oral STS maintenance therapy with quarterly electrolyte and EKG monitoring. Clinical outcomes and adverse events were recorded through January 2026. Table I presents patient characteristics and risk factors, and Table II presents the duration of IV and oral STS therapy and other treatments. Fig 1, Fig 2, Fig 3, Fig 4 show representative photos of each case before, during and/or after treatment (or at the most recent follow-up).

**Table I:** Patient characteristics and risk factors

Case number	Gender	Age at diagnosis	Risk factors
1	Male	67	Hypertension, hyperlipidemia, atrial fibrillation on amiodarone, type II diabetes, obesity, vitamin D deficiency
2	Male	69	Hypertension, coronary atherosclerosis
3	Male	64	obesity, atrial fibrillation on apixaban, alcohol use, hypertension, liver injury, history of DVT, type II diabetes
4	Female	38	Alcohol use, vitamin D deficiency, cirrhosis

**Table II:** Duration of STS treatments and other treatments

Case number	Duration of IV STS treatments	Duration of oral STS treatment	Duration between flares	Other treatments
1	12 wk × 2	58 wk	38 wk	Vitamin D supplementation, Mupirocin 2% ointment + Telfa + Coban wrap
2	12 wk × 2	55 wk	39 wk	Mupirocin 2% ointment
3	12 wk × 3	67 wk	N/A	Mupirocin 2% ointment, Chlorhexidine gluconate 4% wash, Unna boots 10× on LLE and 27 on RLE
4	12 wk	52 wk	N/A	Vitamin D supplementation

## Case 1

A 67-year-old man presented with a chronic, non-healing left lower leg ulcer. Biopsy confirmed epidermal necrosis with peri-eccrine and subcutaneous small vessel calcification consistent with NUC. He received IV STS (25 g twice weekly for 1 month, then 3 times weekly; 36 infusions total), with improvement noted within the first month of receiving IV treatment and complete resolution within 4 months. Eight months later, he experienced a flare and was retreated with a second IV STS course of 36 treatments over 12 weeks, then transitioned to oral STS. Clinical improvement for the second flare was noted at follow-up 8 weeks after re-presentation. He remains flare-free at 12 months, without adverse effects.

## Case 2

A 69-year-old man developed a non-healing left calf wound. Biopsy with direct immunofluorescence results showed ulcer, necrosis, possible superficial fibrin thrombi, and scattered vessels in the subcutis with concentric stippled calcium deposits in the vessel walls and superficial perivascular fibrin deposits, confirming the diagnosis of NUC. He received IV STS (25 g twice weekly for 15 treatments, then 3 times weekly; 36 infusions in total). Clinical improvement in lesions was noted at the follow-up visit, at which point the patient had completed 15 of his 36 scheduled treatments. At completion of 36 treatments, he had complete resolution. Nine months later, a new lesion developed, prompting a second IV STS course of 36 infusions over 12 weeks. Initial improvement was noted at follow-up after completing 18 out of 36 treatments and remission was achieved by completion. He experienced a transient decrease in anion gap but completed therapy and transitioned to oral STS. He remains in remission at 13 months at last follow-up.

## Case 3

A 64-year-old man presented with a painful right thigh wound. Samples sent from surgical debridement showed skin ulceration, severe acute inflammation, dense lymphoplasmacytic infiltrate, granulation tissue, fat necrosis and reactive changes, including septal thickening with inflammation, degenerated collagen fibers with calcification, small capillaries with fine calcium deposit and foci suspicious for intravascular calcification within capillaries, consistent with NUC. He underwent 3 consecutive IV STS courses (25 g 3 times weekly, 36 infusions per course, 108 infusions in total over 36 weeks) due to refractory disease. Notably, the patient experienced progression of his calciphylaxis despite initiation of IV STS treatment 2 months prior. Improvement was first noted at 17 weeks after receiving 53 treatments. At final ulcer resolution, he was transitioned to oral STS, with maintained remission for 12 months as of last follow-up. Adjunctive therapy included weekly Unna boot dressings with topical clobetasol and mupirocin for comorbid venous stasis dermatitis.

## Case 4

A 38-year-old woman presented with multiple painful necrotic, erythematous abdominal to thigh lesions. Incisional biopsy revealed ulcer, hemorrhage, and necrosis of the epidermis, dermis, and subcutis with fibrinoid degeneration of the blood vessel walls and scattered fibrin thrombi with rare concentric and nonconcentric calcium deposits on vessel walls, confirming NUC. She received IV STS (25 g 3 times weekly), but therapy was interrupted for 12 days due to hypokalemia and QTc prolongation. After correcting electrolytes, the QTc normalized, allowing her to complete the course without any subsequent dose reduction. She was then transitioned to oral STS and has remained flare-free after 10 months of follow-up.

## Discussion

NUC is a rare, morbid condition with limited treatment data. In this series, we describe 4 biopsy-confirmed cases that responded to IV STS followed by oral maintenance. All patients remained flare-free at last follow-up, with no significant adverse effects observed during oral therapy.

While IV STS is the most widely reported pharmacologic intervention for calciphylaxis, its efficacy remains uncertain. Recent meta-analyses in patients with chronic kidney disease (CKD) and calciphylaxis have not demonstrated a clear benefit of IV STS for skin lesion improvement or survival and have highlighted the need for randomized controlled trials and better patient selection criteria.^9^ However, these studies have focused almost exclusively on uremic calciphylaxis, and data on NUC remain sparse. Observational studies and case reports, including those by Ning et al and Yu et al, suggest that STS may be effective in non-uremic cases, but standardized protocols and robust outcome data are lacking.^10^^,^^11^ Moreover, these reports do not examine the oral route of administration for initial treatment or preventive maintenance. Our findings suggest that STS may have benefits in non-uremic cases. However, as noted by Abeck et al, who reported successful treatment of NUC using a combination of STS, iloprost, and heparin,^12^ multimodal approaches should also be considered. Triple therapy addresses the 3 hallmarks of calciphylaxis: calcification, vasoconstriction, and microvascular thrombosis. Given the rapid pain relief and complete healing reported, combination treatment may be reasonable for patients who fail STS alone or have severe, uncontrolled pain.

The complexity of NUC is underscored by its hypothesized shared pathophysiology with other ischemic conditions. Hafner proposed that calciphylaxis and Martorell hypertensive ischemic leg ulcers represent different clinical presentations of the same underlying vascular pathology: ischemic subcutaneous arteriolosclerosis.^13^ The conditions share histopathological features and risk factors such as hypertension, diabetes, and vitamin K antagonist use. Because vitamin K antagonists inhibit vitamin K dependent, systemic inhibitors of ectopic calcification, their discontinuation and management of pro-calcific risk factors are central to management.^13^ Recent translational work further highlights inflammatory and thrombotic drivers, including IL-6 upregulation of endothelial tissue factors and activation of the extrinsic coagulation cascade.^14^ This vascular-adipose communication accelerates thrombosis and offers a mechanistic rational for anticoagulant efficacy, as reported by Abeck, and also suggests IL-6 inhibition as a potential therapeutic avenue.

Despite these advances, high-quality clinical trial data remain limited. The CALCIPHYX trial, the first randomized controlled trial in calciphylaxis, evaluated hexasodium fytate.^15^ Although it did not meet its primary endpoints for wound healing or pain reduction, the treatment group demonstrated significantly lower mortality and fewer calciphylaxis-related hospitalizations.^15^ Future studies may assess STS either in comparison with hexasodium fytate using a similar trial design.

The most common side effects of STS include nausea, vomiting, hypotension, headache, disorientation, and prolonged bleeding time. Electrolyte disturbances, including hypokalemia and anion gap (thiosulfate is an unmeasured anion) metabolic acidosis, are also common. Oral STS has a much lower level of systemic absorption; most is oxidized with a small proportion excreted unchanged in urine.^16^ Despite low absorption, we hypothesize that oral STS maintains remission through a sustained-level suppressive effect. While IV STS provides high peak serum concentrations needed to chelate calcium in acute lesions, oral administration may provide sufficient thiosulfate levels to inhibit new nucleation of calcium-phosphate salts in the skin or vasculature. By acting as a steady-state antioxidant and calcium-chelating agent, oral STS may neutralize the pro-calcific environment and improve endothelial nitric oxide peptide signaling, thereby preventing the microvascular occlusion that leads to clinical flares.

Our experience supports the use of IV STS for acute management of NUC, particularly in patients with severe and recurrent disease. Importantly, we observed that compounded oral STS can serve as preventative maintenance therapy. While none of the patients in our series experienced metabolic acidosis or other significant adverse effects on oral therapy, 1 patient experienced hypokalemia and QTc prolongation on IV therapy, consistent with the literature that IV therapy is associated with a higher risk of side effects.^16^ Although both formulations were well tolerated, IV STS adverse effects require close monitoring for metabolic complications and QTc prolongation. In our study, we monitored anion gap and EKG quarterly over the past year when patients were on oral STS and no abnormalities were detected. Therefore, oral STS may not require routine laboratory and EKG monitoring. The drug was well tolerated in our series, with only mild gastrointestinal side effects reported.

The main limitations of our study are its retrospective design, small sample size, and lack of a comparator group. Pain is a key symptom, making the absence of pain-score data a meaningful limitation. Given the rarity of NUC, larger prospective studies and multicenter collaborations will be necessary to better define the role of STS, both IV and oral, in this population. Additionally, the optimal dosing, duration, and monitoring strategies for oral STS remain to be established.

Overall, our findings suggest that a treatment strategy combining IV STS for acute flares and oral STS for preventative maintenance may be effective and well tolerated in patients with NUC. Our patients with recurrent ulceration after initial IV STS at 38 to 39 weeks have not recurred after 52 to 67 weeks of oral therapy. Oral STS may offer a promising maintenance option to reduce relapse risk and minimize treatment-related toxicity. Further studies, including randomized trials, are needed to assess the efficacy, patient selection, and long-term safety of oral STS in NUC.

## Conflicts of interest

None disclosed.

## References

[1] Nigwekar S.U., Thadhani R., Brandenburg V.M. (2018). Calciphylaxis. New Engl J Med.

[2] Ababneh E.I., Hassanein M., Saad A.M. (2022). Calciphylaxis in uraemic and nonuraemic settings: clinical risk factors and histopathological findings. Clin Exp Dermatol.

[3] Zulauf E.E., Connors J.C., Hardy M.A. (2022). Manifestation of nonuremic calciphylaxis in the extremities: case report and review. J Foot Ankle Surg.

[4] Glennon C.M., Xia J., Strowd L. (2025). Outcomes and mortality in calciphylaxis: a multicenter update. J Am Acad Dermatol.

[5] Ross E.A. (2011). Evolution of treatment strategies for calciphylaxis. Am J Nephrol.

[6] Milligan M., Orav E.J., Mitchell A.P., Lam M. (2022). Market determinants of commercial prices for intravenous chemotherapy infusions. J Clin Oncol.

[7] Sivakumar A., Forman H.P., Wang I., Lurie P., Ross J.S. (2025). State policies and facility practices of IV hydration spas in the US. JAMA Intern Med.

[8] AlBugami M.M., Wilson J.A., Clarke J.R., Soroka S.D. (2013). Oral sodium thiosulfate as maintenance therapy for calcific uremic arteriolopathy: a case series. Am J Nephrol.

[9] Wen W., Portales-Castillo I., Seethapathy R. (2023). Intravenous sodium thiosulphate for calciphylaxis of chronic kidney disease: a systematic review and meta-analysis. JAMA Netw Open.

[10] Ning M.S., Dahir K.M., Castellanos E.H., McGirt L.Y. (2013). Sodium thiosulfate in the treatment of non-uremic calciphylaxis. J Dermatol.

[11] Yu W.Y.-H., Bhutani T., Kornik R. (2017). Warfarin-associated nonuremic calciphylaxis. JAMA Dermatol.

[12] Abeck F., Hansen I., Rünger A., Booken N., Schneider S.W. (2024). Successful treatment of non-uremic calciphylaxis with combination therapy with sodium thiosulfate, iloprost, and heparin. Int J Dermatol.

[13] Hafner J. (2016). Calciphylaxis and Martorell hypertensive ischemic leg ulcer: same pattern - one pathophysiology. Dermatology.

[14] Carney E.F. (2025). IL-6 signalling contributes to the pathogenesis of uraemic calciphylaxis. Nat Rev Nephrol.

[15] Sinha S., Nigwekar S.U., Brandenburg V. (2024). Hexasodium fytate for the treatment of calciphylaxis: a randomised, double-blind, phase 3, placebo-controlled trial with an open-label extension. EClinicalMedicine.

[16] Medicine USNLo (2024). Sodium thiosulfate injection, solution. DailyMed.

